# Characterization of X-ray Dose in Murine Animals Using microCT, a New Low-Dose Detector and nanoDot Dosimeters

**DOI:** 10.1371/journal.pone.0049936

**Published:** 2012-11-27

**Authors:** Dustin R. Osborne, Shikui Yan, Alan Stuckey, Lindy Pryer, Tina Richey, Jonathan S. Wall

**Affiliations:** 1 University of Tennessee Graduate School of Medicine, Knoxville, Tennessee, United States of America; 2 Siemens Medical Solutions, Knoxville, Tennessee, United States of America; Northwestern University Feinberg School of Medicine, United States of America

## Abstract

**Background:**

Dose continues to be an area of concern in preclinical imaging studies, especially for those imaging disease progression in longitudinal studies. To our knowledge, this work is the first to characterize and assess dose from the Inveon CT imaging platform using nanoDot dosimeters. This work is also the first to characterize a new low-dose configuration available for this platform.

**Methodology/Principal Findings:**

nanoDot dosimeters from Landauer, Inc. were surgically implanted into 15 wild type mice. Two nanoDots were placed in each animal: one just under the skin behind the spine and the other located centrally within the abdomen. A manufacturer-recommended CT protocol was created with 1 projection per degree of rotation acquired over 360 degrees. For best comparison of the low dose and standard configurations, noise characteristics of the reconstructed images were used to match the acquisition protocol parameters. Results for all dose measurements showed the average dose delivered to the abdomen to be 13.8 cGy±0.74 and 0.97 cGy±0.05 for standard and low dose configurations respectively. Skin measurements of dose averaged 15.99 cGy±0.72 and 1.18 cGy±0.06. For both groups, the standard deviation to mean was less than 5.6%. The maximum dose received for the abdomen was 15.12 cGy and 0.97 cGy while the maximum dose for the skin was 17.3 cGy and 1.32 cGy. Control dosimeters were used for each group to validate that no unwanted additional radiation was present to bias the results.

**Conclusions/Significance:**

This study shows that the Inveon CT platform is suitable for imaging mice both for single and longitudinal studies. Use of low-dose detector hardware results in significant reductions in dose to subjects with a >12x (90%) reduction in delivered dose. Installation of this hardware on another *in vivo* microCT platform resulted in dose reductions of over 9x (89%).

## Introduction

microCT is an important component of the imaging tools available for preclinical researchers because of its ability to generate high-resolution three-dimensional images of the body by using X-rays to create a slice-by-slice reconstruction of the subject being imaged [1]. This process involves using an X-ray source and detector attached to a rotating circular gantry. Typical CT systems use a single poly-energetic X-ray tube with an opposing detector that acquires raw acquisition data as it rotates around the subject [2]. This modality provides the information necessary to determine anatomical structures with the subject and for key data corrections for PET and SPECT data [3].

**Figure 1:**
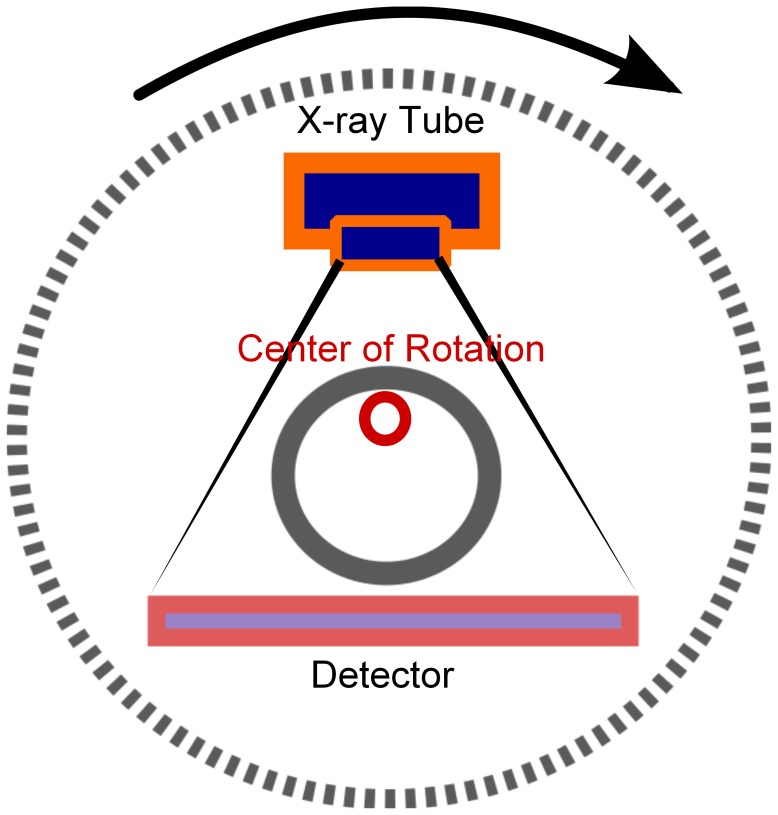
3^rd^ generation detector technology with the X-ray source and detector rotating about the subject.

The Inveon system used in this study uses 3^rd^ generation technology as shown in Figure 1 [4]. It acquires data via a step-and-shoot method meaning that the X-ray source and detector are used to acquire data by rotating to a specified angle and then collecting data at that angle for a specified interval of time called the exposure time. The gantry is then rotated to the next angle and data acquired for the subsequent projections. This set of projection data is then used to reconstruct the final 3D image volume [5]. This particular system also has a continuous rotation step-and-shoot mode to shorten acquisition times but it yields lower-resolution images and was not used in this study. The exposure time, X-ray beam settings (flux) and the number of projections acquired in the image are the primary settings that determine delivered dose during a CT acquisition.

**Figure 2:**
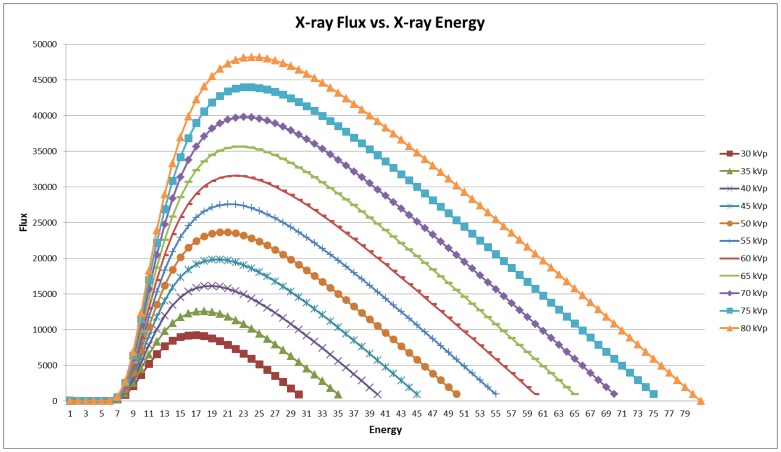
Monte Carlo simulations of Dose vs. X-ray energy (keV).

Dose is the amount of energy absorbed per unit mass (J/kg) in the subject on which the beam is incident [6]. If the delivered dose is too great, the incident X-rays may cause physiological changes in the subject or the disease such as inhibiting/causing tumor growth [7]. The flux of an X-ray tube is proportional to the square of the voltage settings and directly proportional to the current settings of the tube as well as the exposure time at each projection [8]. Figure 2 shows a plot of X-ray flux versus X-ray energy (keV) (Siemens Medical Solutions) derived from a manufacturer supplied dose estimator.

Some manufacturers of preclinical CT systems have focused their design efforts on high resolution imaging. Many commercially available microCT systems require relatively high X-ray exposure because their manufacturers optimize for resolution instead of dose. Dose from high resolution protocols is often greater because the settings for high resolution applications usually result in extended subject exposure to X-rays [9]. There are few publications from preclinical manufacturers with regard to the dose delivered for typical protocols and often the only information available are those estimates provided by the vendor. This lack of information makes planning for longitudinal studies more difficult in preclinical research as information on the delivered dose for a given setting is critical to assessing potential biological effects of X-ray dose on the study [10]–[12].

**Figure 3:**
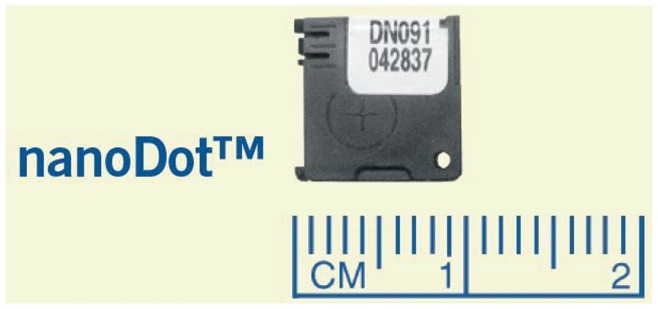
nanoDot dosimeter technology with scale to illustrate size.

This study used a new generation of dosimeters, called nanoDots™, from Landuaer Inc. These dosimeters are shown in Figure 3 and offer a wide range of detection from 5 keV to >20 MeV with accuracies of ±2% with the screened variation of this dosimeter. The nanoDot series are flat and have a small area (∼1 cm^2^) with a serial number and bar code printed on each individual dosimeter, enabling easier tracking throughout the study. This technology is ideal for dose measurements of this type as they should not have an angular or energy dependence that biases the results (Landauer, Inc. nanoDot Data Sheet).

This study aims to be the first to determine the dose characteristics of the Inveon CT imaging system (Siemens Medical Solutions) in mice. Although, much of the basic CT hardware is similar to the microCAT II platform, it is possible that minor variations in dose characteristics could be seen from previous work characterizing the microCAT and microCAT II platforms[12]–[16]. This work will provide a reference for Inveon CT users regarding the dose performance of this system to aid in planning of longitudinal experiments on this imaging platform.

This study also seeks to characterize the dose performance of this platform using a new low-dose configuration available from the manufacturer. This includes examination of the primary hardware component of this low-dose technology on another CT system to examine the dose-savings on other microCT platforms. This new hardware uses an optimized scintillator material to convert the incident X-rays to visible light. Per the manufacturer, this technology improves the overall sensitivity of the system to X-rays by up to a factor of 5 and improves measured signal to noise ratio by up to a factor of 12 (Siemens Medical Solutions, USA, Inc.).

The increased sensitivity and signal to noise characteristics makes it possible to reduce the exposure time per projection and X-ray source current, thus lowering the total dose delivered during a microCT acquisition. With this particular hardware, increases in sensitivity and signal to noise come at the expense of a slight decrease in maximum resolution. According to preliminary manufacturer’s tests using a 10 µm wire phantom, the measured reconstructed image resolution for the low magnification settings in the standard configuration was approximately 130 µm FWHM while the resolution for the low dose configuration at low magnification was measured to be approximately 210 µm FWHM.

## Materials and Methods

### Dosimeters

The dosimeters selected for this study were a relatively new technology from Landauer Inc., called nanoDots. These particular dosimeters were selected because they are specified by the manufacturer to have a lower limit of detection of 0.1 mGy with a useful energy range of 5 keV –20 MeV. This matched the range of energies and doses expected in this study. The manufacturer also indicates that their design is such that they experience no angular or energy dependence thus making it a good candidate for dose measurements of this nature.

Landauer Inc. offers the options of purchasing pre-screened dosimeters or standard dosimeters. The standard nanoDot measures with a manufacturer reported accuracy of ±10% (k = 2). Screening of the dosimeters provides details of the reproducibility of that individual dosimeter and enables selection of those dosimeters with better measurement accuracy. Screened dosimeters give a manufacturer reported accuracy of ±5% (k = 2). Both the standard and screened variations are specified to give a precision of ±5% (k = 2).

The Screened nanoDot dosimeters were ordered for this study as they provide improved accuracy in dose measurement. Each dosimeter was individually serialized and bar coded. The serial numbers for each dosimeter were recorded in spreadsheet software along with information on the location of the dosimeter in which it was surgically implanted. The nanoDots were separated into 2 groups: 1 to measure skin dose and the other to measure dose in the abdomen. Each group had a dedicated control dosimeter kept isolated from any sources of radiation to verify that no other radiation sources in the lab affected the study. Figure 3 shows an image of the nanoDot dosimeter.

### Animal Model

**Figure 4:**
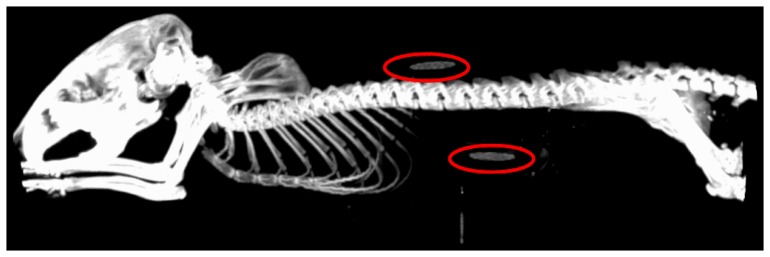
Back and abdominal dosimeter placement within the subject.

All animal experiments were post-mortem and performed in an AAALAC-I-accredited institution under the auspices of an Institutional Animal Care and Use-approved protocol (#1628) approved by the University of Tennessee Knoxville Institutional Animal Care and Use Committee (UTK-IACUC). The carcasses were shared from this protocol in accordance with procedures outlined in the Guide for the Care and Use of Laboratory Animals. Each mouse had 2 nanoDot dosimeters surgically implanted with 1 dosimeter located centrally in the abdomen and the other placed at the base of the neck just below the skin. These locations were chosen to give a range of maximum and minimum dose-depths, and to have enough axial separation to minimize potential scatter effects, while also attempting to maintain equivalent X-ray exposure on each nanoDot during the acquisition. Figure 4 shows a MIP from the reconstructed CT data with the locations of the dosimeters highlighted.

### Imaging

All CT workflows were performed on the CT subsystem of an Inveon Trimodality (PET-SPECT-CT) system equipped with a variable focal spot X-ray source. The CT protocol used in this study was designed to represent a scan that might be typical for whole-body mouse imaging. The detector settings used the full field of view with a detector binning factor of 4 applied, resulting in 512 × 768 projections and 512 × 512 × 768 reconstructed images.

The imaging protocol for the standard detector used X-ray settings of 80 kVp at 0.5 mA with a 0.5 mm aluminum filter. The exposure time was set using the recommendations for ideal exposure from the manufacturer instruction manual. It directs the user to examine a histogram of the counts recorded on the detector and use the exposure time and X-ray beam settings to move the histogram to the recommended count level. On our system, and with the beam settings listed above, this resulted in an exposure time of 250 ms per projection. The data were acquired over 360 degrees using a manufacturer recommended protocol with 1 projection acquired per degree of rotation.

**Table 1:** Descriptive statistics for group 1 using the standard CT detector.

Descriptive Statistics for Standard Dose Configuration			
*Group 1 N = 5*			
	Average ± StDev	Maximum	Minimum
**Abdomen**	13.48±0.97	15.12	12.70
**Skin**	15.35±0.41	15.84	14.94
**Group 1 Totals**	14.42±1.22	15.84	12.70

**Table 2:** Descriptive statistics for group 2 using the standard CT detector.

Descriptive Statistics for Standard Dose Configuration			
*Group 2 N = 10*			
	Average ± StDev	Maximum	Minimum
**Abdomen**	13.97±0.74	14.71	12.77
**Skin**	16.31±0.62	17.3	15.62
**Group 2 Totals**	15.14±1.33	17.3	12.77

Following completion of the standard configuration dose measurements, the low-dose detector hardware was installed and the acquisition protocol modified to prevent over-exposure of projections due to the system’s heightened sensitivity to X-rays. The protocol used to assess the low-dose hardware kept the same X-ray voltage and sampling settings as the standard-configuration measurements (80 kVp with 360 projections acquired over 360 degrees). But in order to better compare the low-dose and standard configurations, the X-ray current and exposure time settings were adjusted to affect a statistically equivalent signal-to-noise ratio as calculated by means equivalence analysis described below. The matched SNR was achieved with an exposure time of 50 ms and an X-ray current setting of 0.2 mA indicating an exposure time reduction of ∼78% (4.5x) and a decrease in X-ray current of ∼60% (2.5x).

**Table 3:** Descriptive statistics for all groups, all dosimeters using the standard CT detector.

Descriptive Statistics for Standard Dose Configuration			
*All Dosimeters, All Groups*			
Average ± StDev (cGy)	Maximum (cGy)	Minimum (cGy)	Means Equivalent
14.9±1.32	17.3	12.70	Yes, p<0.05

This work includes two analyses of data where it is critical to show the statistical equivalence of mean values between two independent groups. When research questions demand statistical evidence to demonstrate the similarity of two groups (as opposed to difference) employing equivalency testing procedures is more appropriate than traditional statistical testing. To assess the baseline similarities between groups, a series of Two One-Sided *t*-test (TOST) equivalency procedures were conducted [17].

The TOST procedure is an alternative method for conducting and interpreting independent *t*-tests where the researcher *a priori* defines an acceptably trivial difference between the two groups, which would signify the two as statistically equivalent. This acceptable margin is then applied to the test to discern whether the two groups are statistically significantly equivalent to one another. For the present study, each set of groups were tested to ensure that their combined (**average recorded dose and SNR**) were statistically equivalent using the FDA guidelines of 80%–125% as the maximum tolerable shift between the ratio for the log transformed differences in means of the two samples. Using this approach arguably provides additional protection against erroneously concluding that statistically significant differences exist when faced with small sample sizes such as in the present study. All TOST procedures and TOST power analyses were performed using the Equivalence and PowerTOST packages available for the R statistical software package [18].

**Table 4:** Average SNR values calculated for each subject. Averages for each configuration are given.

SNR Measurement using Logarithmic Formulation 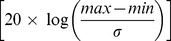						
	Subject 1	Subject 2	Subject 3	Subject 4	Subject 5	SNR Average
**Standard**	16.89	16.96	16.46	17.29	16.76	16.87
**Low- Dose**	16.30	15.86	15.82	15.96	15.83	15.95
**SNR Measurement using Standard Formulation 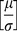 **						
	**Subject 1**	**Subject 2**	**Subject 3**	**Subject 4**	**Subject 5**	**SNR Average**
**Standard**	13.98	16.59	14.12	12.08	13.64	14.08
**Low-Dose**	17.60	15.05	16.89	16.10	18.05	16.74

Noise characteristics of the data were matched by measuring regions of interest in air, brain, bone and liver that were drawn in the reconstructed data from the standard and low-dose configurations. A spherical region of 4.2 mm^3^ was used to measure statistics in the liver, brain and air. For bone measurements, a global threshold was applied to Hounsfield Unit (HU) calibrated data to create a region of interest on each dataset that consisted of voxel intensities ranging from 1500–8000 HU.

Signal to noise ratios were calculated for each region using the following formulations:
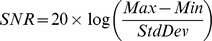


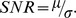
SNR measurements for each region were averaged and the results for the low-dose configuration were compared to the baseline data acquired using the standard detector hardware. The current and exposure time settings of the low-dose protocol were then adjusted accordingly until the SNR values were statistically matched using the Two One Sided Test (TOST) procedure. Power analysis was performed to obtain the number of observations required to show means equivalence between the two configurations.

**Table 5:** Descriptive statistics for group 1 using the low-dose detector.

Descriptive Statistics for Low-Dose Assessment			
*Group 1 N = 5*			
	Average ± StDev	Maximum	Minimum
**Abdomen**	1.03±0.03	1.08	1.00
**Skin**	1.19±0.02	1.22	1.17
**Group 1 Totals**	1.11±0.09	1.22	1.00

**Table 6:** Descriptive statistics for group 2 using the low-dose CT detector.

Descriptive Statistics for Low-Dose Configuration			
*Group 2 N = 10*			
	Average ± StDev	Maximum	Minimum
**Abdomen**	0.94±0.03	0.97	0.88
**Skin**	1.17±0.07	1.32	1.04
**Group 2 Totals**	1.06±0.13	1.32	0.88

A comparison of image quality was performed by imaging samples using both the low-dose and standard configurations at manufacturer recommended settings and the settings for the low-dose protocol. The manufacturer recommended protocol incorporated X-ray beam settings of 80 kVp, 0.5 mA and exposure times of 250 ms and 50 ms respectively for the standard and low-dose configurations. The low-dose protocol used X-ray beam settings of 80 kVp, 0.2 mA and an exposure time of 50 ms. Data were acquired using these protocols and descriptive statistics calculated for the same regions of interest drawn for the SNR equivalence analysis.

All projection data were reconstructed in real-time using a networked reconstruction workstation and the COne Beam Reconstruction Algorithm (COBRA™) software by Exxim Computing Corporation. Beam-hardening correction was used in the reconstruction using the manufacturer’s default parameter values. The reconstruction protocol for each detector configuration was also was calibrated to Hounsfield Units (HU) per manufacturer instructions. Reconstruction downsampling was disabled to prevent unnecessary loss of resolution and smoothing of the data that may bias the comparison.

**Table 7:** Descriptive statistics for all dosimeters, all groups using the low-dose CT detector.

Descriptive Statistics for Low-Dose Assessment			
*All Dosimeters, All Groups*			
Average ± StDev (cGy)	Maximum (cGy)	Minimum (cGy)	Means Equivalent
1.07±0.12	1.32	0.88	Yes, p>0.05

To further test the low-dose technology described in this work, the primary component of the low-dose hardware was removed from the Inveon CT and installed on our microCAT II+SPECT imaging system. This is a 2^nd^ generation CT system from Imtek that also included their 1^st^ generation SPECT detector design. This system has is an *in vivo* imaging unit similar to the Inveon with an X-ray tube and detector mounted orthogonally on a rotating stage.

Dose measurements were performed on this platform using a 50 cc centrifuge tube filled with water and four nanoDot dosimeters fixed to the walls of the tube. The dosimeters were arranged orthogonal to one another with two dosimeters at each end of the area imaged. The locations of the dosimeters were marked on the tube to enable exact repositioning of dosimeters between scans. Similar to the study performed on the Inveon platform, noise measurements in a water phantom were used to determine the appropriate protocol settings between the standard and low-dose configurations.

Although, other acquisition parameters remained the same, the X-ray tube settings on the micoCAT configuration differed from the Inveon with the baseline scan using a voltage of 70 kVp and current of 0.5 mA. The “ideal” exposure, based on manufacturer recommendations resulted in an exposure time of 300 ms. The low-dose protocol used X-ray tube settings of 70 kVp with a current of 0.19 mA. The exposure time required to match the noise in the baseline scan was 80 ms.

**Figure 5:**
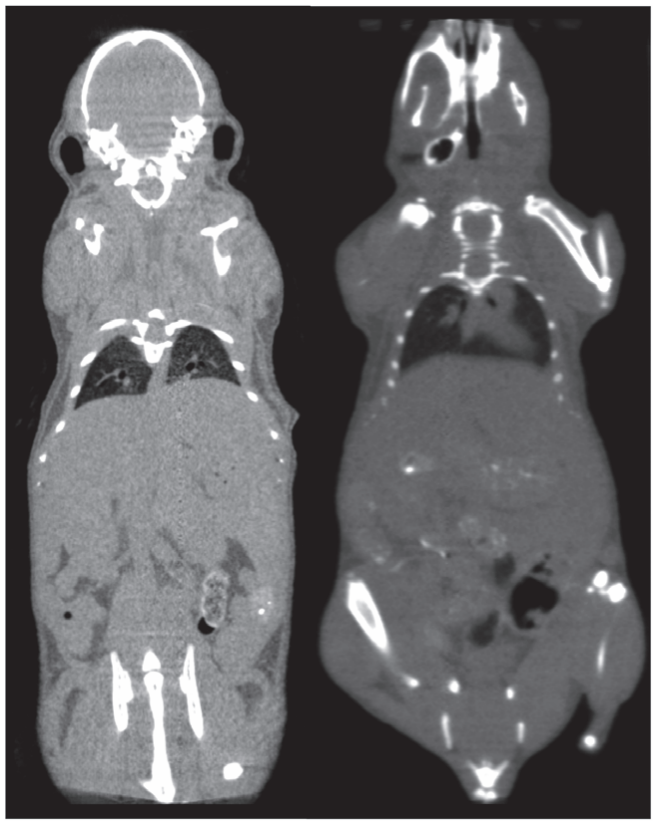
Representative data from standard (left) and low-dose (right) acquisitions using beam settings of 80 kVp, 0.5 mA and an exposure time of 250 ms and 50 ms for each configuration, respectively.

**Figure 6:**
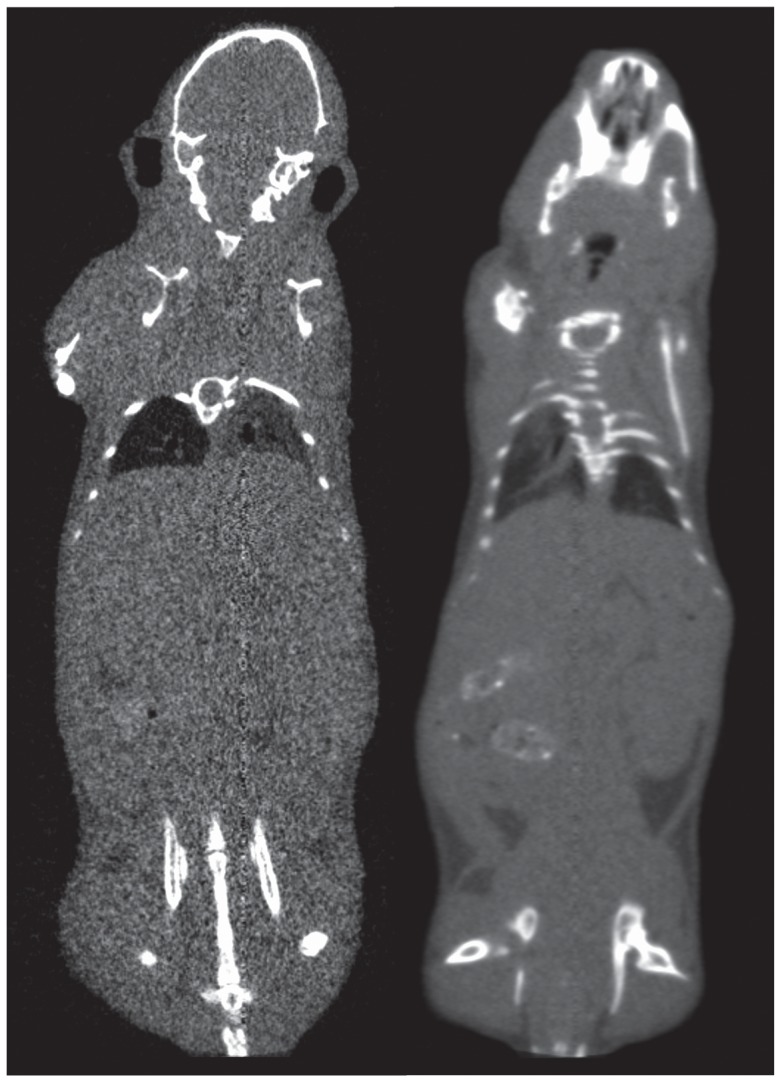
Images from the standard (left) and low-dose (right) configurations acquired at low-dose settings of 80 kVp, 0.2 mA and 50 ms exposure time.

### Study Design

A preliminary water phantom study was performed to provide initial information on the expected change in dose between the standard and low dose configurations. This information was used to make an assessment regarding the number of animals that would be required for each population in order to be statistically significant. Preliminary studies indicated at least a 12x reduction in dose with a standard deviation assumed to be no more than 5 cGy among the population.

G power statistical software was used to run Wilcox-Mann-Whitney power tests to assess the required sample size to show a statistical difference between samples [19], [20]. These tests indicated that to reach an α of 0.05 with a 95% confidence interval, at least 9 samples for each configuration was needed for a total population size of 18 animals. A 95% confidence interval with an α of 0.01 would require a sample size of 24 animals. Using this information, a total of 30 animals were used to achieve a high quality statistical comparison between the data. Although the difference in dose between the standard and low dose configurations was significant, t-tests were performed to statistically validate this difference.

This post-mortem dose study was performed beginning with an initial pilot study of 5 animals followed by an additional 10 animals upon assessment of initial results. The dosimeters were implanted into the sacrificed mice using the smallest possible incisions at each of the areas of interest. The dosimeters were implanted orthogonal to the beam and the incision closed. The animals were placed onto individual cardboard strips that were serialized for each mouse in the study. The animals were then placed onto the 38-mm carbon-fiber pallet provided with the Inveon scanner and the imaging protocol was then executed for each animal in the study.

**Table 8:** Direct comparison of the standard and low-dose configuration results.

Descriptive Statistics for Both Detector Configurations				
N = 15				
	Average Dose (cGy)	Standard Deviation (cGy)	Maximum Dose (cGy)	Minimum Dose (cGy)
Standard	14.9	1.32	17.32	12.70
Low-Dose	1.07	0.12	1.32	0.88

Following imaging, the dosimeters were removed and again verified against the recorded serial numbers and expected locations. The dosimeters and control dosimeters were returned to their individual zip-seal packaging and placed into a shipping envelope. The envelope was returned to Landauer, Inc. for processing along with radiation information required by Landauer, Inc. to calibrate and quantify the dosimeter measurements. Measurement results were received within 3 business days.

An identical study was repeated approximately 5.5 months after the initial 5 mice were completed. The second group of animals consisted of 10 wild type mice imaged with both the standard and low-dose detector configurations. These animals were prepared and imaged in the same manner and under identical conditions to the first imaging study.

## Results and Discussion

### Standard Configuration Dose Measurements

Results showed the average dose delivered to the abdomen for groups 1 and 2 to be 13.48 cGy±0.97 cGy and 13.97±0.74 cGy respectively. Dose to the skin for groups 1 and 2 measured an average of 15.35 cGy±0.41 cGy and 16.31±0.62. For both abdomen and skin measurements, the standard deviation to mean was less than 5.35%. The maximum dose received in the abdomen for both groups was 15.12 cGy while the maximum dose for the skin was 17.30 cGy. Minimum doses for the abdomen and skin were 12.7 cGy and 14.94 cGy respectively. These results are all listed in Tables 1 and 2.

The mean dose for all dosimeters used with the standard detector configuration was 14.9 cGy±1.32 cGy and a median dose of 15.02 cGy. On average the skin dose from incident X-rays was 15.8% greater than that recorded in the abdomen. Control dosimeters were kept with each group to validate that no additional radiation was present during each phase of the study that could add bias to the results.

**Table 9:** Estimations of dose using additional protocol optimization techniques.

Inveon Standard Configuration Dose Estimations					
Projections	Exposure Time (ms)		kVp	Current (µA)	Estimated Dose (cGy)
360	225		80	500	14.9
220	225		80	500	9.3
**Inveon Low Dose Configuration Dose Estimations**					
360		50	80	200	1.07
220		50	80	200	0.67
**Dose Estimate for Standard Configuration Based on Optimized Protocols ** [**16**]					
360		56	80	500	5.6

Because two groups of data were acquired on separate days, a group of 5 the first day followed by 10 additional mice on the second day, statistical tests were performed to validate that the means between the two groups were statistically equivalent. TOST analysis of the data yielded p = 6.7e-5 indicating the mean values were equivalent (p<.05). Post hoc power analysis of the data revealed that 100% power was obtained with the number of samples being compared. Using the FDA “80–125” guidelines and with the low coefficient of variation seen between the two samples, power analysis indicated that n = 4 in each group would have been sufficient to reach 99% power. Table 3 shows descriptive statistics for all samples from the standard configuration population and the results of the means equivalence testing.

An examination of previous dose assessments performed on the similar microCAT imaging platform (Siemens Medical Solutions USA, Inc.) indicates that the delivered dose is very similar but tends to be lower on the newer Inveon platform. Measurements on the Inveon platform had a maximum dose value of 17.3 cGy for the skin where previous dose assessments have shown the microCAT line of scanners to have maximums on the order of 19–22 cGy [11], [13]. Other work using the microCAT platform referenced in this paper had significant variation in values for dose ranging from 2.8–9.0 cGy, however, the experimental conditions were different, either in the beam settings or in the type of instrumentation used to measure the dose. Additional variation could be because of individual dosimeter placement.

### Reduction of Dose between Configurations

The low-dose configuration for this imaging platform resulted in significant reductions in delivered dose compared to the standard configuration. Measurements between the two configurations showed an average reduction of 13.9x (93%). Even though the percentage reduction was significant, t-tests were performed to quantify the statistical significance of the difference for completeness. Welch Two Sample t-tests resulted in clear indications that the two groups of data were significantly different with p<<0.05 (p = 2.2e-16). Post-hoc power tests indicated that with 15 samples from each group a power of 100% was obtained.

### Signal-to-Noise Ratio Comparison

The average signal to noise ratios for images acquired using the standard configuration was 16.87 and 14.08 for the logarithmic and standard SNR formulations respectively. The low-dose SNR averages were 15.95 and 16.74 for the respective formulations. SNR averages across both configurations for the log and standard formulations were 16.41 and 15.41 respectively. Table 4 shows the recorded values for average SNR for each subject and each method of SNR calculation.

Means equivalence testing of the SNR results yielded p-values of p = 4.9e-7 and p = 0.018, for the log and standard formulations respectively, indicating statistically equivalent means (p<0.05). Power analysis of the TOST procedure indicated that our measurement of average SNR from 5 samples with a population coefficient of variation of 1.2% and 4.7% for each calculation of SNR respectively, achieved a power of >90%.

### Low-Dose Configuration Dose Measurements

Dose measurements for the low-dose configuration indicated the average dose delivered to the abdomen for both groups to be 1.03±0.03 cGy and 0.94±0.03 cGy, respectively. Dose to the skin measured an average of 1.19±0.02 cGy and 1.17±0.07 cGy. For both groups, the standard deviation to mean was less than 5.6%. The maximum dose received for the abdomen was 1.08 cGy while the maximum dose for the skin was 1.32 cGy. Minimum doses for the abdomen and skin were 0.88 cGy and 1.04 cGy respectively. Tables 5 and 6 show the calculated statistics for the two low-dose groups.

The mean dose for all dosimeters used with the standard detector configuration was 1.07 cGy±0.12 cGy and a median dose of 1.06 cGy. On average, the skin dose from incident X-rays for the subjects imaged with the low dose configuration was 22% greater than that recorded in the abdomen. Control dosimeters were also kept with each low-dose dosimeter group to validate the absence of contamination from external radiation sources.

As with the standard configuration, the subjects in the low-dose population were imaged in two groups on separate days. The first group consisted of 5 animals with the second group consisting of 10 additional wild type mice imaged approximately 5.5 months after the first group. The same TOST procedure described above was applied to this data to show means equivalence. Means were determine to be statistically equivalent with p = 6.33e-8 indicating equivalence for (p<0.05). Post-hoc power analysis for the TOST procedure for this group resulted in >90% power for at least n = 5 in each group. Table 7 shows the descriptive statistics calculated for all measurements in the low-dose population as well as the results of means equivalence testing.

The measurements performed with the low-dose technology on the microCAT II+SPECT platform yielded results similar to that seen on the Inveon with the low-dose protocol resulting in an average dose of 1.1 cGy±0.02 cGy. Standard protocol measurements resulted in an average dose of 10.45 cGy±0.23 cGy. This indicates a reduction in dose of just over 9x (89%) showing a significant reduction in dose on this platform as well. The primary difference in the overall dose reduction results between the microCAT and Inveon platforms could be attributed to several factors, including differences between the scanner hardware on the Inveon that enable a slight improvement in overall dose reduction compared to the microCAT reduced sensitivity of the CT detector because of age and model or differences in the reconstruction software version.

Both sets of results for the Inveon and microCAT CT platforms indicates that doses of less than 1 cGy could be achieved with optimized protocols such as using a half-scan or exposure times that are less than those recommended by the manufacturer. The low-dose measurements of approximately 1 cGy compare to other *in vivo* imaging systems currently available such as the IVIS Spectrum CT and Quantum FX CT system that provide manufacturer reported doses of 1 cGy or less.

### Image Quality Comparison

Visual inspection of images from the low dose and standard configurations using full X-ray tube power (80 kVp @ 0.5 mA) with ideal exposure showed an overall decrease in resolution for the data acquired using the low-dose detector. Images acquired in the standard configuration appear to be much sharper and more detailed. Images from the standard configuration showed increased noise compared to the low-dose images because of the decreased signal to noise ratio performance for the standard configuration when compared to the low-dose hardware. Representative images can be seen in Figure 5.

Quantitative comparisons of image quality for the data acquired using the low-dose settings of 80 kVp, 0.2 mA and a 50 ms exposure time showed significant image quality differences between the two configurations. Reconstructed data for the standard detector showed significantly more noise with a corresponding decrease in overall contrast compared to the low-dose data acquired at the low power settings. The images acquired using the low-dose and standard detector resulted in an average standard deviation of 178 HU and 348 HU across all regions of interest. The average SNR (µ/σ formulation) resulted in values of 3.3 and 16.7 for the standard and low-dose detector configurations indicating an 80% (5x) decrease in SNR for the standard configuration versus the low-dose hardware. Figure 6 shows representative images from each configuration acquired at the low-dose settings.

This work gives dose data that enables better assessment of delivered dose in the planning of preclinical imaging studies on the Inveon CT platform. This work, to our knowledge, is also the first to use the nanoDot line of dosimeters for a preclinical dose assessment study, as well as the first to characterize the performance of a new lowdose configuration for this imaging platform. With this quantitative assessment of dose for a given CT protocol and the knowledge of how X-ray flux behaves with respect to each of the key user-controlled X-ray tube and detector settings, it is possible to better approximate the dose delivered at other X-ray settings. The placement of the dosimeters both within the abdomen and just below the surface of the skin gives Inveon users an idea of the range of doses that can be expected at the minimum and maximum dose depositions within the mouse. Table 8 shows a comparison of average dose values and standard deviations between the 2 detector configurations for all acquisitions.

Future work in this area could more comprehensively study beam settings compared to estimates of dose based on the proportionalities of the X-ray settings and the flux. Although this information would be useful, the number of parameters that are user-selectable on the Inveon platform would make it difficult to fully characterize the dose performance using every possible setting. Previous studies, such as those by Osborne, et. al., have shown optimizations of scan protocols as one possible way to achieve balance between dose and image quality characteristics [21]. Using the dose-optimized protocols from that work, we estimate that abdomen and skin doses on the Inveon CT platform could be on the order of 6–10 cGy for the standard configuration and significantly reduced doses on the order of 0.5 cGy for the low-dose option. These estimates are outlined in Table 9.

Previous work from Laforest and Daibes regarding dose effects in murine tumor models, [11], [14], suggest that there is a minimal therapeutic effect for X-ray radiation doses of 6–8 cGy. These works also have indications of reduced tumor growth rates at these dose levels. Laforest noted a 63% smaller tumor sample compared to controls for daily dose delivery of 6 cGy, and 73% smaller tumor samples with 22 cGy daily doses. Laforest showed tumors receiving higher doses of 11 cGy and 55 cGy took days longer to reach the control tumor size of 95 mm^3^. Daibes’ results for a B16F10 tumor model indicate that animals receiving up to 5 sequential X-ray doses of 7–8 cGy resulted in negligible mean survival rates (28.7±4.2 days control, 30.6±3.4 days treated).

This suggests that the dose rates for the protocol used in this work could have some potential for tumor growth inhibition. This can be potentially mitigated by a number of methods such as using dose-optimized protocols that reduce the risk of affecting tumor growth [16]. The low-dose hardware in combination with protocol optimization techniques shows the greatest reduction in dose on this particular imaging platform.
